# Dietary amino acids and incidence of hypertension: A principle component analysis approach

**DOI:** 10.1038/s41598-017-17047-0

**Published:** 2017-12-04

**Authors:** Farshad Teymoori, Golaleh Asghari, Parvin Mirmiran, Fereidoun Azizi

**Affiliations:** 1 Nutrition and Endocrine Research Center, Research Institute for Endocrine Sciences, Shahid Beheshti University of Medical Sciences, Tehran, I.R. Iran; 2 Endocrine Research Center, Research Institute for Endocrine Sciences, Shahid Beheshti University of Medical Sciences, Tehran, I.R. Iran

## Abstract

The current study aimed to investigate the association between dietary amino acid patterns and incidence of hypertension, using principal components factor analyses. This study was conducted within the framework of Tehran Lipid and Glucose Study on 4288 adults, who were free of hypertension at baseline (2008–2011) and were followed for three years (2011–2014). Principal component factor analyses were conducted based on eight amino acid groups and three amino acid patterns were extracted. The first pattern was characterized by branched chain, aromatic, and alcoholic amino acids, and proline. Acidic amino acids and proline were highly loaded in the second pattern and the third was characterized by sulphuric and small amino acids. Adjusted odds ratio of the highest quartile of the first pattern was 1.83 (95%CI: 1.21–2.77, P for trend = 0.002) compared to the lowest one. The first pattern had high positive correlation with dietary intakes of animal protein and dairy, but was negatively correlated with plant protein, fruit, and vegetable. There was no significant association for the second and third patterns. Findings indicate that the dietary amino acid pattern, rich in branched chain, aromatic, and alcoholic amino acids, and proline could increase the risk of hypertension.

## Introduction

The association between dietary protein intake and blood pressure (BP) has recently been receiving considerable attention^1–3^. It is thought that amino acids as protein components and their dietary sources of different food groups may be responsible for some previously reported relations between dietary protein and chronic diseases^4,5^. Several studies have investigated the association of different amino acids with BP and reported inconsistent results^4,6–9^. Glutamic acid in the INTERMAP and female twin cross-sectional studies has been reported to have an inverse relation with BP^4,9^, whereas two cohort studies showed no association with hypertension^7,8^. Similarly, glycine indicates very different results in two cross-sectional studies^4,6^, controversies which have also shown for tyrosine, methionine, and alanine^4,7,8^.

Since individual amino acids are not consumed alone and have interactions with other amino acids, it is probable that higher intakes of several amino acids simultaneously increase or decrease the risk of hypertension. Recently, several studies used nutrient pattern analyses to investigate possible associations between diet components and chronic disease^10–13^. The nutrient pattern approach is a combination of several nutrients derived from nutritional data to better reflect probable underlying mechanisms of the diet and disease relationship^11^.

To the best of our knowledge there is no study available examining dietary amino acid patterns with hypertension. To test whether different amino acid groups could be both positively and negatively related to hypertension, we aimed to identify dietary amino acid patterns, using principal component analysis in participants of the Tehran Lipid and Glucose Study (TLGS) population and determine the association of dietary amino acid patterns with incidence of hypertension.

## Material and Methods

### Design and study population

Our study was conducted within the framework of the TLGS, a population-based prospective study aimed at determining the risk factors of non-communicable diseases among 15005 subjects aged ≥3 years, residents of district 13 of Tehran, the capital of Iran^14^. This ongoing study was begun in March 1999 and data are collected at three-year intervals; the baseline survey, a cross-sectional study, was conducted from 1999 to 2001, and surveys 2 (2002–2005), 3 (2006–2008), 4 (2009–2011), and 5 (2012–2014) were prospective follow-up surveys. From among 12823 participants in the fourth survey (2009–2011) with complete data of medical history and physical examination, 7956 were randomly selected for this dietary assessment.

For the current study, a total of 6493 individuals with complete dietary data, aged 20 to 70 years, from the fourth survey were enrolled and followed through the fifth survey as an outcome examination (median follow-up: 3.1 years). After exclusion of subjects with under- or over-reported dietary intakes (<800 kcal/d or >4500 kcal/d, respectively) or those on specific diets (n = 317), those with history of myocardial infraction, cerebral vascular accidents, or cancers (n = 43), those with hypertension (n = 1057), lactating and pregnant women (n = 106), 5004 subjects were followed up. Some individuals fell into more than one exclusion category. Of 5004 participants followed through the fifth survey (2012–2014), 716 subjects who had missing data on BP in the follow-up assessment were excluded, leaving 4288 participants for the final analysis (Follow up rate: 86.2%).

The ethics committee of the Research Institute for Endocrine Sciences of Shahid Beheshti University of Medical Sciences approved the study protocol and all participants provided written informed consent. All experiments were performed in accordance with the Declaration of Helsinki as well as our institutional guidelines.

### Dietary assessment

To evaluate the usual dietary intakes of participants, a valid and reliable semi-quantitative food frequency questionnaire (FFQ)^15^, was used by trained dieticians with at least 5 years of experience in the TLGS survey. The interviewer read out the food items on the FFQ, and recorded participants’ consumption in portion sizes and frequency during the previous year on a daily, weekly, or monthly basis, which took about 45 minutes for each participant. Daily intakes of each food item were determined based on the consumption frequency multiplied by the portion size or household measure for each food item, data, which were then converted to grams. Portion sizes of consumed foods, which were reported in household measures, were specified according to the US Department of Agriculture (USDA) standard portion sizes (e.g., apple, 1 medium; bread, 1 slice; dairy, 1 cup). When unable to use the USDA portion sizes, household measures (e.g., beans, 1 tablespoon; chicken meat, 1 leg or wing; rice, 1 large or small plate) were used alternatively. Energy and nutrient intakes were calculated using USDA food composition table (FCT) and for local food items not included in FCT we used the Iranian food composition table.

Amino acid intake was calculated using the United States Department of Agriculture (USDA National Nutrient Database for Standard Reference, Release 28, 2015) food composition table (available on http://www.ars.usda.gov/ba/bhnrc/ndl), which is based on the chemical analysis of amino acid composition^16^. We categorized amino acids based on chemical structures into eight groups including branched chain, aromatic, alkaline, sulfuric, acidic, alcoholic, and small amino acids and proline^1^.

### Physical activity assessment

Assessment of physical activity levels was conducted using a Modifiable Activity Questionnaire (MAQ), modified and validated previously among Iranians^17^; the frequency and time spent during the past 12 months on activities of light, moderate, hard, and very hard intensity, according to a list of common activities of daily life were obtained from all individuals; metabolic equivalent task (MET) hours per week (MET-h/wk), as physical activity levels criteria was calculated for all participants.

### Clinical and biological measurements

Using pretested questionnaire data on socio-demographics, anthropometrics, medical history of chronic disease (cancer, stroke, hypertension, cardiovascular disease, or diabetes), medication use, and smoking habits were collected via interviews by trained interviewers. Weight measurement was done using digital scales with an accuracy of up to 100 grams, while subjects were without shoes or socks and wore light clothing. Height was measured using a stadiometer with accuracy of 0.5 cm for participants, without shoes, in a standing position, with shoulders in normal alignment. Body mass index (BMI) was calculated as weight in kilograms, divided by height in meters squared (kg/m^2^). WC measurement was conducted by unstreched shape tape meter to the nearest 0.1 cm accuracy at the level of the umbilicus, over light clothing, and without any pressure.

Blood pressure, at baseline and at the end of follow-up, was measured twice on the right arm, with a minimum interval of 30 seconds, after resting for at least 15 minutes, sitting on a chair using a mercury sphygmomanometer and the Korotkoff sound technique, with an accuracy of 2 mm Hg; the average of two measurements was considered as the subjects’ final pressure; systolic blood pressure (SBP) was determined with the onset the first sound heard and diastolic blood pressure (DBP) with disappearance of the sound.

Blood samples were taken from all study participants after 12–14 hr overnight fasting, between 7:00 and 9:00 a.m at baseline. All the blood analyses were done by Selectra 2 auto-analyzer (Vital Scientific, Spankeren, Netherlands) and commercial kits (Pars Azmoon Inc., Tehran, Iran) at the TLGS research laboratory on the day of blood collection. Fasting plasma glucose (FPG) was measured by the enzymatic colorimetric method using glucose oxidase. For all subjects who were not on anti-diabetic drugs, the standard 2 h serum glucose test was performed. HDL-C was measured after precipitation of the apolipoprotein B-containing lipoproteins with phosphotungstic acid and for serum triglycerides (TGs) measurement, an enzymatic colorimetric method with glycerol phosphateoxidase was used.

### Definition

To define hypertension, separately for subjects aged above and below 60 years, JNC8 criteria were used^18,19^. Hypertension was defined as SBP ≥140, or DBP ≥90, or taking antihypertensive medications in subjects, aged <60 years and SBP ≥150, or DBP ≥90 or taking antihypertensive medications in subjects, aged >60 years. Diabetes was defined based on the American Diabetes Association (ADA) criteria as FBS ≥126 mg/dl or 2-h post 75 gram plasma glucose ≥200 mg/dl or use of anti-diabetic drugs for definite diagnosis of diabetes^20^. Smoking status included smokers (subjects who smoke cigarettes daily or occasionally) and non-smokers.

### Statistical analyses

Statistical analyses were done using Statistical Package for Social Sciences (version 15.0; SPSS Inc, Chicago IL). Dietary amino acid patterns were derived by principal component analysis with varimax rotation and based on the correlation matrix. Statistical correlation between variables and adequacy of sample size was tested, using the Bartlett test of sphericity (*P* < 0.001) and the Kaiser-Mayer-Olkin test (0.60). We extracted three factors based on scree plot (eigenvalue > 1) and factor scores for all participants for each of the three extracted factors were calculated by summing the frequency of consumption, multiplied by factor loadings across all amino acid group items. Baseline characteristics of participants for continuous variables and categorical variables are presented as mean ± SD or median (25–75 interquartile range), and percentages, respectively. To test the trend of qualitative and quantitative variables across quartiles of each dietary amino acid patterns score (median value), Chi-square and linear regression was used, respectively. Logistic regression was used to analyse the relation between dietary amino acid pattern and risk of hypertension incidents after three years of follow up. Odds ratio (OR) and 95% confidence interval (CI) are reported. Models were adjusted for age, sex, diabetes, BMI, physical activity, smoking, and daily energy intake. Additional adjustments were made for saturated fatty acids (SFA), polyunsaturated fatty acids (PUFA), monounsaturated fatty acids (MUFA), fiber, calcium, magnesium, sodium, and potassium. All P values are two sided, and those below 0.05 were considered to be statistically significant.

## Results

**Figure 1:**
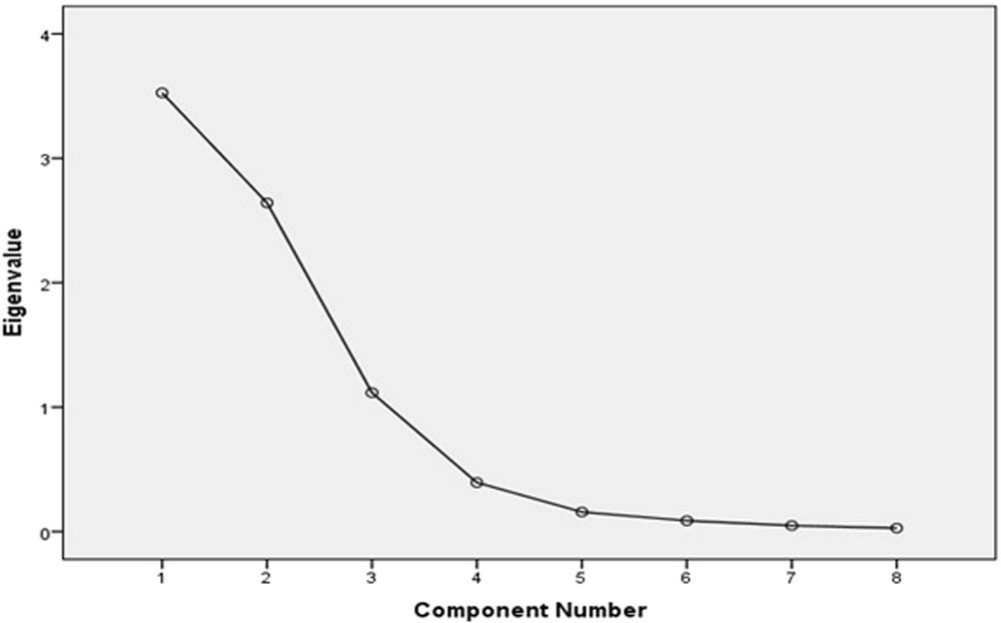
Scree plot for extraction of dietary amino acid patterns by principal component analysis. Percentage of amino acids from total protein was combined into eight amino acid groups and used as input variables. Factors with eigenvalues >1 were identified amino acid dietary pattern.

The mean (SD) of participants (41.9% males) age and BMI were 39.7 (12.8) years and 26.9 (4.6) kg/m^2^, respectively. After three years of follow up, 429 (10%) cases of incident hypertension were documented. As percentage of total protein, glutamic acid was the most predominant dietary amino acid (21.85 ± 1.78) and tryptophan the least (1.15 ± 0.07). Eighteen amino acids were categorized into eight groups based on amino acid structures (Table 1). Three major dietary amino acid patterns were extracted using factor analyses with eigenvalues > 1 from the scree plot (Fig. 1). The factor-loading matrix for dietary amino acid patterns is presented in Table 2. The three patterns explained 91% of the total variation in dietary intakes of eight amino acid groups. The first pattern (eigenvalue = 3.5) was characterized by branched chain, alcoholic, and aromatic amino acids, and proline, and was negatively loaded for small amino acids. The second pattern (eigenvalue = 2.6) was characterized by acidic amino acids and proline, and negatively loaded for alkaline and small amino acids. The third pattern (eigenvalue = 1.1) was characterized by sulfuric and small amino acids.

**Table 1:** Amino acid groups used for factor analyses.

Amino acid group	Amino acids included
Branched chain amino acids	Leucine, isoleucine, and valine
Aromatic amino acids	Tryptophan, phenylalanine, and tyrosine
Alkaline amino acids	Histidine, arginine, and lysine
Sulfuric amino acids	Methionine and cysteine
Acidic amino acids	Glutamic acid and aspartic acid
Alcoholic amino acids	Serine and threonine
Small amino acids	Glycine and alanine
Cyclic side chain amino acid	Proline

**Table 2:** Factor-loading matrix for 3 major dietary amino acid patterns identified by principal component analysis*.

	Factor 1	Factor 2	Factor 3
Branched chain amino acids	0.95	—	—
Aromatic amino acids	0.93	0.21	0.2
Alkaline amino acids	0.33	−0.88	—
Sulfuric amino acids	0.32	—	0.89
Acidic amino acids	—	0.87	—
Alcoholic amino acids	0.95	—	—
Small amino acids	−0.31	−0.64	0.62
Proline	0.55	0.76	—
Cumulative variance explained (%)^†^	41.6	74.8	91.0

^*^Absolute values <0.20 are not shown for simplicity and easy interpretation.

^†^Percentage of variance in total amino acid groups intake is explained by patterns.

Baseline characteristics of study participants according to amino acids dietary patterns are shown in Table 3. The frequency of men and dietary intake of energy, carbohydrate, plant protein, PUFA, sodium, magnesium and fiber decreased (*P* < 0.05) whereas HDL-C, and dietary intakes of total and animal protein, total fat, SFA, MUFA, potassium, and calcium increased (*P* < 0.05) across quartiles of the first pattern. Subjects were more likely to be males, older, smokers and had higher dietary intakes of energy, plant protein, carbohydrate, sodium, magnesium and fibre (*P* < 0.05), as well as lower HDL-C and dietary intakes of total fat, SFA, MUFA, PUFA, total protein, animal protein, potassium, and calcium (*P* < 0.05) according to increasing quartiles of the second pattern. Across quartiles of the third pattern, subjects were more likely to be men, older, had higher physical activity levels, serum level of TGs, and dietary intakes of sodium, carbohydrate, total, animal and plant protein (*P* < 0.01); however, dietary intakes of energy, total fat, SFA, MUFA, PUFA, potassium, calcium, magnesium, fiber, serum HDL-c and BMI decreased (*P* < 0.01).

**Table 3:** Baseline characteristics of participants across quartiles of amino acid patterns in the Tehran Lipid and Glucose Study.

Characteristics	Pattern 1		Pattern 2		Pattern 3	
	Q 1	Q 4	Q 1	Q 4	Q 1	Q 4
Age (years)	39.6 ± 12.1	40.1 ± 11.8	39.1 ± 12.2	40.4 ± 12.2^†^	40.9 ± 12.1	37.4 ± 11.7^*^
Men (%)	41.9	35.5^*^	35.4	51.6^*^	27	54.2^*^
Diabetes (%)	5.3	5.1	5.6	4.4	6.3	4.9
Body mass index (kg/m^2^)	27.0 ± 4.6	27.2 ± 4.5	26.9 ± 4.8	27.0 ± 4.5	27.3 ± 4.6	26.6 ± 4.5^†^
Smoking (%)	11.6	10.5	12.9	13.0^‡^	6.9	14.4
Systolic blood pressure (mmHg)	108.9 ± 11.9	108.9 ± 12.2	108.5 ± 11.7	109.3 ± 12.2	108.5 ± 12.5	109.0 ± 11.9
Diastolic blood pressure (mmHg)	73.3 ± 8.4	73.2 ± 8.6	72.9 ± 8.2	73.4 ± 8.5	73.0 ± 8.2	73.4 ± 8.5
Physical activity (MET.h/week)	62.4 (25.9–93.9)	58.0 (23.8–89.3)	58.6 (28.1–90.7)	62.5 (21.4–96.7)	54.5 (23.8–85.2)	68.0 (31.8–99.2)^*^
High density lipoprotein cholesterol (mg/dl)	47.6 ± 11.6	48.6 ± 11.9^‡^	48.8 ± 11.7	46.7 ± 11.2^*^	49.6 ± 11.9	47.0 ± 11.2^*^
Triglycerides (mg/dl)	114 (81–166)	116 (80–167)	109 (78–159)	117 (82–171)	108 (78–153)	117 (80–171) ^‡^
Dietary intakes						
Energy (kcal)	2538 ± 789	2276 ± 727^*^	2413 ± 779	2530 ± 754^*^	2504 ± 790	2383 ± 747^*^
Total protein (% of energy)	13.2 ± 3.4	14.4 ± 2.6^*^	14.9 ± 3.6	13.3 ± 1.7^*^	13.1 ± 3.0	14.4 ± 2.7^*^
Plant protein (% of energy)	7.3 ± 2.0	6.1 ± 1.4^*^	6.2 ± 2.0	7.9 ± 1.4^*^	6.4 ± 2	7.1 ± 1.5^*^
Animal protein (% of energy)	5.8 ± 3.0	8.2 ± 2.1^*^	8.7 ± 3.3	5.3 ± 1.7^*^	6.6 ± 2.3	7.3 ± 3.3^*^
Total fat (% of energy)	28.3 ± 6.3	31.4 ± 6.0^*^	31.4 ± 6.6	28.8 ± 5.5^*^	32.1 ± 6.6	28.0 ± 5.9^*^
Saturated fatty acids (% of energy)	8.1 ± 2.3	11.4 ± 2.7^*^	10.1 ± 2.8	9.07 ± 2.5^*^	10.6 ± 3.1	8.8 ± 2.4^*^
Monounsaturated fatty acid (% of energy)	9.4 ± 2.6	10.3 ± 2.6^*^	10.5 ± 2.8	9.3 ± 2.3^*^	10.2 ± 2.8	9.6 ± 2.5^*^
Polyunsaturated fatty acids (% of energy)	5.9 ± 2.0	5.8 ± 1.9^‡^	6.3 ± 2.2	5.6 ± 1.6^*^	6.1 ± 2.2	5.8 ± 1.9^†^
Carbohydrates (% of energy)	61.5 ± 7.0	56.0 ± 5.8^*^	55.8 ± 6.9	61.4 ± 5.8^*^	57.7 ± 7.7	59.3 ± 6.4^*^
Sodium (mg/1000 kcal)	1542 ± 479	1496 ± 407^‡^	1461 ± 492	1644 ± 403^*^	1477 ± 455	1530 ± 423^†^
Potassium (mg/1000 kcal)	1960 ± 595	2019 ± 520^†^	2027 ± 566	1796 ± 502^*^	2259 ± 610	1658 ± 401^*^
Magnesium (mg/1000 kcal)	199 ± 43	194 ± 37^†^	189 ± 40	209 ± 42^*^	201 ± 43	189 ± 39^*^
Calcium (mg/1000 kcal)	519 ± 186	757 ± 194^*^	610 ± 213	589 ± 182^‡^	710 ± 236	518 ± 155^*^
Fiber (g/1000 kcal)	21.6 ± 6.9	17.7 ± 7.2^*^	17.3 ± 5.6	22.3 ± 8.4^*^	20.4 ± 6.6	17.9 ± 6.4^*^

^*^P for trend <0.001; ^†^<0.01; ^‡^<0.05.

Table 4 presents the association between each dietary amino acid pattern and incidence of hypertension after three years of follow up; in the fully adjusted model, the OR of the highest quartile score of the first pattern was 1.83 (95% CI: 1.21–2.77, *P* for trend = 0.002), compared to the lowest. For the second and third patterns of dietary amino acids intake, no significant association with incident hypertension was found, although the third pattern did have a slight tendency to reduce the risk of hypertension [OR = 0.81; 95% CI: 0.65-1.16, *P* for trend = 0.20].

**Table 4:** Odds ratios (95% confidence interval) for incidence of hypertension across quartile scores of dietary amino acid patterns.

Dietary amino acid pattern		Quartiles			P for trend
	Q1	Q2	Q3	Q4	
**Pattern 1**					
**Hypertension/total**	97/1072	100/1072	115/1072	117/1072	
Model1*	1.00 (Ref)	1.05 (0.77–1.42)	1.30 (0.97–1.74)	1.25 (0.93–1.67)	0.07
Model2^†^	1.00 (Ref)	1.08 (0.79–1.47)	1.38 (1.02–1.86)	1.24 (0.92–1.68)	0.08
Model3^‡^	1.00 (Ref)	1.24 (0.90–1.72)	1.72 (1.21–2.44)	1.83 (1.21–2.77)	0.002
**Pattern 2**					
**Hypertension/total**	107/1072	93/1072	109/1072	120/1072	
Model1*	1.00 (Ref)	0.88 (0.65–1.19)	1.03 (0.77–1.38)	1.01 (0.75–1.34)	0.71
Model2^†^	1.00 (Ref)	0.88 (0.65–1.20)	1.05 (0.78–1.41)	1.00 (0.74–1.34)	0.76
Model3^‡^	1.00 (Ref)	0.91 (0.67–1.25)	1.10 (0.81–1.50)	1.04 (0.74–1.48)	0.60
**Pattern3**					
**Hypertension/total**	119/1072	119/1072	101/1072	90/1072	
Model1*	1.00 (Ref)	1.01 (0.76–1.34)	0.88 (0.66–1.18)	0.83 (0.61–1.12)	0.17
Model2^†^	1.00 (Ref)	1.09 (0.81–1.45)	0.92 (0.68–1.24)	0.83 (0.61–1.14)	0.20
Model3^‡^	1.00 (Ref)	1.10 (0.81–1.48)	0.90 (0.65–1.25)	0.81 (0.65–1.16)	0.20

^*^Adjusted for age and sex.

^†^Adjusted for model 1 and diabetes, body mass index, physical activity, smoking (yes or no) and daily energy intake.

^‡^Adjusted for model 2 and saturated fatty acids, poly unsaturated fatty acids, mono unsaturated fatty acids, calcium, magnesium, sodium, potassium, and fiber (all continuous).

The partial correlation of dietary amino acid patterns with food sources and dietary nutrient are summarized in Table 5. There was a significant moderate positive correlation between the first pattern and total animal source foods, dairy, animal protein, total fat, and SFA, whereas the first pattern conversely correlated with total plant source foods, fruit and vegetable, and plant protein. The second pattern was positively correlated with grains, plant protein, but had an inverse correlation with red meat, fish, poultry, and egg, nuts, and animal protein. The partial correlation of the third pattern was positive with fish, poultry, egg, and grain, total protein and selenium but was negative with dairy, fruit and vegetable calcium and potassium.

**Table 5:** Partial correlation coefficient of dietary amino acid patterns with food sources and nutrients.

	Pattern 1	Pattern 2	Pattern 3
Food sources*			
Total animal source foods	0.670^†^	−0.153^†^	−0.192^†^
Dairy (g/d)	0.707^†^	−0.025	−0.291^†^
Red meat (g/d)	−0.077^†^	−0.393^†^	0.168^†^
Processed and organ meat (g/d)	0.086^†^	−0.032^‡^	0.210^†^
Fish, poultry, and eggs (g/d)	−0.123^†^	−0.524^†^	0.335^†^
Total plant source foods (g/d)	−0.219^†^	−0.020	−0.219^†^
Grains (g/d)	−0.122^†^	0.329^†^	0.483^†^
Legumes(g/d)	0.036^‡^	−0.152^†^	−0.183^†^
Nuts(g/d)	−0.003	−0.254^†^	−0.037^‡^
Fruit and vegetable(g/d)	−0.303^†^	−0.118^†^	−0.374^†^
Nutrients intake**			
Total protein (% of energy)	0.199^†^	−0.278^†^	0.228^†^
Animal protein (% of energy)	0.379^†^	−0.528^†^	0.150^†^
Plant protein (% of energy)	−0.293^†^	0.410^†^	0.118^†^
Total fat (% of energy)	0.209^†^	−0.203^†^	−0.216^†^
Saturated fatty acids (% of energy)	0.482^†^	−0.152^†^	−0.209^†^
Monounsaturated fatty acid (% of energy)	0.129^†^	−0.164^†^	−0.029
Polyunsaturated fatty acids (% of energy)	−0.042^‡^	−0.122^†^	−0.008
Total trans (% of energy)	−0.012	−0.024	−0.012
Sodium (mg/1000 kcal)	−0.024	0.195^†^	0.074^†^
Calcium (mg/1000 kcal)	0.428^†^	−0.013	−0.300^†^
Potassium (mg/1000 kcal)	0.006	−0.148^†^	−0.355^†^
Magnesium (mg/1000 kcal)	−0.063^†^	0.187^†^	−0.081^†^
Phosphorus (mg/1000 kcal)	0.518^†^	0.070^†^	−0.045^‡^
Iron (mg/1000 kcal)	−0.079^†^	−0.036^‡^	−0.112^†^
Copper (mg/1000 kcal)	−0.166^†^	0.052^†^	−0.025
Selenium (mg/1000 kcal)	−0.067^†^	0.483^†^	0.334^†^

^*^Adjusted for age, sex, body mass index, and energy intake.

^**^Adjusted for age, sex, and body mass index.

^†^P < 0.001.

^‡^P < 0.05.

## Discussion

In the current prospective analysis of the TLGS, we extracted three dietary amino acid patterns using principal component analyses method for assessing the relationship between dietary amino acids intakes and the risk of hypertension (Fig. 1, Table 2). The first dietary amino acids pattern identified by branched, alcoholic, and aromatic amino acids, and proline, was associated with increased risk of hypertension incidents. Although all amino acid patterns did not show significant associations with hypertension, the third pattern characterized by small and sulfuric amino acids had the tendency to reduce the risk of hypertension (Table 4).

To our knowledge this is the first study examining the association between dietary amino acid patterns and incidence of hypertension. However, several individual amino acids previously studied, documented contradictory findings on their associations with blood pressure. In the INTERMAP study, dietary phenylalanine, which was mostly supplied from plant sources showed no association with BP^9^, whereas phenylalanine and valine intakes were related to higher BP^8,21^ similar to our first pattern, which is highly loaded with these amino acids. Tyrosine has been found to be associated with lower BP in cross sectional studies^4,7^, although, cohort studies show no association of tyrosine with BP^7^. Although arginine as the precursor of NO had lowering effects on BP in trial studies^22,23^, conflicting results were reported by epidemiological ones^1,6,7,21^. These discrepancies in individual amino acid studies may be explained by the design, sample size, and different groups in these studies. Another possible explanation is the consumption of different protein sources and different amounts of individual amino acid intakes from various sources, e. g. in the INTERMAP and Rotterdam study^7,9^, glutamic acid was the most predominant amino acid in the diet similar to ours, whereas in the Norouzi Javidan *et al*. study, lysine was the predominant dietary amino acid and glutamic acid had low intakes^21^.

Since amino acids consumed together have interactions and synergistic effects, it is probable that examining individual amino acids may not yield accurate results. Recently, pattern analysis, a complementary approach introduced to examine the diet-disease relationship provides a holistic picture of nutrient consumption and disease relation and overcomes limitations such as biological interaction between food and nutrients^24,25^ and since protein-constituent amino acids are no exception, previous studies indicate conflicting results on associations between individual amino acids and BP. Therefore, assessing dietary amino acid patterns may provide a deeper insight into possible relationships between amino acids and hypertension. Our study for the first time has identified three patterns and examined the relationship of patterns with the incidence of hypertension.

As shown in Table 4, the first pattern which was highly loaded by BCAAs, AAAs, and alcoholic amino acids increased the risk of incident hypertension after adjusting for potential confounders. Elevated plasma levels of BCAAs and AAAs increased risk of chronic diseases such as hypertension via probable intermediate metabolites^26^. As shown in Table 5, the first pattern had high positive correlation with total animal food sources and dairy, and moderate negative correlation with a range of healthy foods including fish, poultry, grains, fruit and vegetables; it was also positively correlated with animal protein and SFA and negatively correlated with plant protein, which previously indicated adverse and protective relations with blood pressure, respectively^27–29^.

As previously mentioned, source of amino acids is a determinant factor in the relation of amino acids and disease. In our study, 55% of total amino acids in the first factor was consumed from animal sources, which was 10% higher than plant sources. Animal protein sources also constituted more amount of each of the BCAAs, AAAs and alcoholic amino acids. Therefore, it is probable that higher intakes of BCAAs, AAAs and alcoholic amino acids from animal sources in the first pattern have synergistic effects, augmenting mutually effects of each other, eventually increasing the risk of incident hypertension. This was confirmed by a recent study of our investigation group, i.e. higher serine intakes increased the risk of hypertension incidents by 70%^30^. Intake of alcoholic amino acids (serine and threonine) classified as a dietary pattern besides BCAAs and AAAs increased the risk of hypertension to 83% by interactions and synergistic effects among these three groups of amino acids.

Such an effect about amino acid sources on disease incidence was indicated previously, e.g, BCAAs in two recent studies (with similar design and follow-up time) have documented totally contradictory findings^5,31^; in the former, in Japan, the predominant dietary sources of BCAAs were cereals, potatoes, and starches (23–25%), followed by fish and shellfish (21–23%) and meats (14–15%), which reduced the risk of diabetes by 30%^31^; however, in the United States, BCAAs were mainly obtained from meat (37%), followed by fish (8%) and dairy (12%), which increased risk of diabetes by 12%^5^. In our study, BCAAs were mostly supplied by dairy products (31.5%), cereals (29%), meats (20.5%), and fish (3.4%). Similarly, in our study, AAAs were received from animal sources (56%), whereas in the INTTERMAP study, these were mostly received from plant sources^9^.

A variety of biochemical mechanisms may explain the findings observed for the first pattern; biochemical metabolites of dietary AAAs can increase the BP via activating the sympathetic system and increasing the vascular tone of vessels^32^ and putrefactive products of AAAs can also have adverse effects on BP by thickening the vessels^33^. BCAAs and their metabolites can also affect blood pressure through adverse effects on insulin resistance^34^, similar to the relation of insulin resistance and hypertension identified previously^35^. Higher serum levels of BCAAs and serine can eventually reduce the tryptophan and threonine or glutamic acid entrance into the brain, respectively, and consequently decrease the synthesis of BP-beneficial neurotransmitters^36,37^.

The second pattern which was highly loaded by acidic amino acids and proline showed no significant association with incident hypertension in the current study (Table 4). Despite the inconsistency in the glutamic-BP relationship in epidemiologic studies^7,9,21^, it is expected that glutamic acid have beneficial effects on BP, by contributing to synthesis of glutathione as a potent antioxidant in the body, and being a substrate of arginine, a nitric oxide precursor^9^. However, this pattern was negatively correlated with arginine and histidine that have previously been associated with lower BP^4,23^. It is probable that the beneficial effect expected due to glutamic acid on hypertension in the second pattern, was counteracted by the strong detrimental effect of arginine and histidine in this pattern, which is why this pattern did not show significant results.

Although not significant, as indicated in Table 4 the third pattern tended to reduce the risk of hypertension. This pattern had a positive correlation with consumption of fish, poultry, grain, total protein and selenium, and was negatively correlated with consumption of fruit, vegetable, dairy, total fat, SFA, calcium, and potassium (Table 5).

Previous studies have reported that methionine and alanine increase the risk of high BP^1,8^; however, findings on glycine are inconsistent^4,6^. Interestingly, cysteine and glycine lowers BP by contributing to synthesis of glutathione and binding with excess aldehydes^7,38^. As well, in analysis of glycine intake with incidence of hypertension, we observed a non-significant inverse association in all participants, however in subjects, aged ≥30 years a significantly lower risk (up to 31%) for hypertension was found when comparing the highest quartile of glycine vs the lowest; there is also a protective relationship between dietary cysteine and hypertension in this age group. However, it seems that the combined pattern of sulfuric and small amino acids in our study, attenuates the individual amino acid power, which results in non-significant reductions for the risk of hypertension; this finding indicates that the interaction effect between dietary amino acids in terms dietary amino acids pattern in relation to hypertension deserves to be tested in future cohort studies.

Of the current study’s strengths, for the first time, we used the principal component analyses method to introduce dietary amino acid patterns. Furthermore, prospective design and a large sample size within a population-based study allowed casual inference and generalization of findings to the whole population. In addition, collection of nutritional and clinical data by trained interviewers, rather than individual self-reports increased the validity of our results. Finally, sensitivity analyses showed that after exclusion of smokers and subjects with extreme BMI or diabetes, results remained significant (data not shown). The limitations of our study are: first, despite adjusting of a wide variety of variables, the confounding effect of some unknown and unmeasured residual confounders may have occurred. Furthermore, lack of measurement of serum amino acids restricted us from better interpretation of the associations observed between dietary amino acid patterns and risk of hypertension.

In conclusion, our data suggest that a dietary amino acid patterns which are mainly characterized with BCAAs, AAAs, alcoholic amino acids, and proline are associated with higher risk of incident hypertension. A holistic view of total amino acids presents better and more realistic findings about the association of amino acids and hypertension. Our findings have important clinical implications; more prospective studies are definitely required to investigate this association in other populations and detect the beneficial and detrimental effects of related amino acid patterns on chronic disease.
